# Photo-inactivation of *Escherichia coli* and *Enterococcus hirae* using methylene blue and sodium anthraquinone-2-sulphonate: effect of process parameters

**DOI:** 10.1007/s13205-016-0487-6

**Published:** 2016-08-20

**Authors:** Madhavi Singh, Kannan Pakshirajan, Vishal Trivedi

**Affiliations:** Department of Biosciences and Bioengineering, Indian Institute of Technology Guwahati, Guwahati, 781039 Assam India

**Keywords:** Photo-inactivation, Methylene blue, Sodium anthraquinone-2-sulphonate, *E. coli*, *E. hirae*, Flow cytometry

## Abstract

In this study, effect of different parameters, viz. concentration of photosensitizer (PS), pH of the bacterial cell suspension and initial cell count, on photo-inactivation of *Escherichia coli* and *Enterococcus hirae* bacteria using methylene blue (MB) and sodium anthraquinone-2-sulphonate (SAQS) was investigated employing the statistically valid full factorial design of experiments. The inactivation efficiency of *E. hirae* using MB ranges between 10.81 and 48.55 %, whereas in the case of *E. coli* it ranges between 10.41 and 46.44 %. Using SAQS, the inactivation efficiency of *E. hirae* was within 5.26–39.03 %, and in the case of *E. coli* it varied in the range 4.65–37.66 %. Statistical analysis of the photo-inactivation results in the form of analysis of variance (ANOVA) and student ‘*t*’ test revealed significant individual effect of these process parameters. In addition, an increase in dark incubation period with MB or SAQS resulted in enhanced photo-inactivation efficiency against both the microorganisms. Reactive oxygen species measurement and analysis of lipid peroxidation and protein carbonyl index helped in a better understanding of the photo-inactivation mechanism.

## Introduction

Photosensitization is an emerging technique for efficient disinfection of drinking water and tertiary treated wastewater due to its easy implementation and cost effectiveness. It involves the combination of an active substance, i.e., a photosensitizer, light and molecular oxygen to achieve the purpose. This method is slowly taking over the existing conventional disinfection methods like chlorination, UV and ozone due to the drawbacks associated with these latter techniques. For instance, chlorination, the most widely used disinfection method suffers from the formation of disinfection by-products which have carcinogenic and mutagenic effects on mammals (Marugan et al. 2010; Pablos et al. 2011). On the other hand, ozone the most powerful disinfectant among chemical disinfectants is not feasible due to its escape from the water during the application thereby posing threat to the health of operators and the environment even at a concentration as low as 0.03 gm^−3^ (Acher et al. 1997), whereas UV treatment is considered costly and its exposure causes mutations.

During the last one decade a large number of photosensitizers have been tested in vitro and in vivo mainly focussing on their antimicrobial efficiency in relation to the structure of these compounds (Luksiene 2005). Photosensitizers on absorbing visible light enter into its excited state and can effect electron transfer reactions (Redox reactions) (Type I), also direct transfer of energy to ground state oxygen forms toxic singlet oxygen (Type II) which are responsible for bacterial inactivation (Wainwright and Crossley 2004). Few studies have reported that Gram positive bacteria are susceptible to any kind of dye, cationic, anionic or neutral, whereas Gram negative bacteria show resistance due to the presence of lipopolysaccharide coat that presents a physical and chemical barrier to singlet oxygen species produced outside the cells, which must enter the cell to interact with vital targets such as membrane or cytoplasmic components in order to inactivate these bacteria (Dahl et al. 1989). To render these bacteria susceptible to photo-inactivation, permeability of their outer membrane can be increased by some pre-treatment step using either chemical or biological agents (Valduga et al. 2004). On the other hand, cationic dyes are found efficient in inactivating both the kinds of bacteria (Alves et al. 2009) but are in general more efficient against Gram positive bacteria (Ergaieg and Seux 2009). Some studies have also been conducted on the effect of simple parameters like concentration of photosensitizer by Ergaieg and Seux (2009) which showed an increase in inactivation kinetics with increase in photosensitizer concentration whereas a study by Chen et al. (2011) showed alkaline pH of microbial suspension to be more effective for inactivation under visible light; however, to our knowledge, no investigation has been carried out focussing on the combined effect of these parameters which is essential for a better understanding of the process as well as for its successful scale up applications.

This work, therefore, aimed to study the combined effect of concentration of photo sensitizer, pH of the cell suspension and viable cell count employing the statistically valid full factorial design of experiments. It is expected that all the three parameters will show some significant combined effects on bacterial inactivation. Also the generation of reactive oxygen species (ROS) and its effect on bacteria is studied. The bacterial strains used in this photo-inactivation study were *E. coli* and *E. hirae*; the photosensitizers tested were methylene blue (MB) and sodium anthraquinone-2-sulphonate (SAQS). *E. coli* and *E. hirae* are known as non-pathogenic indicator microorganisms whose presence in water indicates the possible presence of pathogenic microorganisms. *E. coli* is indicator of fecal contamination whereas *E. hirae* is indicator of contamination due to surface runoff.

## Materials and methods

### Chemicals and reagents

The photosensitizers (PS), methylene blue (MB) and sodium anthraquinone-2-sulphonate (SAQS) used in this study were purchased from Sigma Aldrich, India. These photosensitizers were chosen due to their cationic nature. Bacterial growth media, such as nutrient broth, brain heart infusion broth and agar were procured from Merck (India). Analytical reagents propidium iodide, dihydrochlorofluorescin diacetate (DCFDA), tri carboxylic acid (TCA), guanidine hydrochloride and 2,4-dinitrophenylhydrazine (DNPH) were purchased from Sigma Aldrich, India and other chemicals thiobarbituric acid (TBARS) and 1,1,3,3-tetraethoxy propane were obtained from Merck (India).

### Microorganisms and culture conditions

The bacterial strains *Escherichia coli* (MTCC 1610) and *Enterococcus hirae* (MTCC 3612) were obtained from IMTECH, Chandigarh, India. *E. coli* was grown in nutrient broth, whereas *E. hirae* was cultured using brain heart infusion broth both at 37 °C, 180 rpm for 24 h.

### Inactivation experiments

Batch experiments in this study were carried out as per the statistically valid 2^3^ full factorial design by varying the concentration of PS, pH of the bacterial suspension and initial cell count as shown in Table 1. The initial concentration level (0.73 µmol/l) of the PS used was based on a study by Ergaieg and Seux (2009) whereas the pH level was kept in the basic range to simulate real wastewater pH which is mostly alkaline. Dilution range adopted was decided based on a preliminary experiment carried out in our laboratory without any PS. Each parameter level was coded as −1, 0 and +1 to represent low, center and high level, respectively. Combination of the parameters and their levels used in these experiments is presented in Table 2.

**Table 1:** Range and levels of the variables used in the photo-inactivation experiments

Variables	Low level (−1)	Center point (0)	High level (+1)
Concentration of PS (µmol/L)	0.73	0.99	1.25
pH	7.50	8.25	9.00
Dilution	10	100	1000

**Table 2:** Combination of parameters and their levels used in the photo-inactivation experiments

Experimental run no.	Coded levels of the variables		
	PS initial concentration (µmol/L)	pH	Dilution
1	−1	−1	−1
2	−1	−1	+1
3	−1	+1	−1
4	−1	+1	+1
5	0	0	0
6	+1	−1	−1
7	+1	−1	+1
8	+1	+1	−1
9	+1	+1	+1

All the inactivation experiments were carried out in triplicate by transferring 1 mL of 24 h grown bacterial culture (*E. hirae* or *E. coli*) into 1.5 mL Eppendorf tube and centrifuging the biomass at 10,000×*g* for 10 min. The pellets obtained were washed twice with phosphate buffer saline (PBS) of respective initial pH and re-suspended in PBS having different initial pH 7.5, 8.25, 9.0, as per the design (Table 1) followed by serial dilution up to 1000 times. PBS composition used was 8 g/L NaCl, 0.2 g/L KCl, 1.44 g/L Na_2_HPO_4_ and 0.25 g/L KH_2_PO_4_ and its pH was adjusted using 0.1 N HCl and 0.1 N NaOH. From these PBS suspended cultures a set of control experiments were carried out without addition of MB and SAQS for counting initial CFU and the other suspended cultures were added with MB and SAQS from their respective 1 mM stock solution to ensure initial concentration in the range of 0.73–1.25 µmol/L as per the experimental design (Table 2). One set of mixtures was kept in dark for 30 min and the other set of mixtures was kept under dark condition on a gel rocker platform for three different incubation periods of 5, 15 and 30 min with constant shaking. For MB mediated inactivation, a commercially available 11 W compact fluorescent lamp (CFL) with a light intensity of 2700 lx in a closed chamber was used as the light source. The exposure period was 10 min and the emitted light wavelength was well within the visible light range (400–700 nm), which correlated well with the MB absorbance wavelength. In case of SAQS mediated inactivation in this study, a commercially available 6 W Philips™ blacklight blue tube with IEC value of 0.88 W and a light intensity of 20 lx in a closed chamber was used as the UV-A source which matched with the photosensitizer absorbance wavelength. Control experiments carried out using these respective light sources and either only the photosensitizers or the bacteria revealed negligible effect on both. Bacterial suspensions (10 µL) from all sets of mixtures were then spread on brain heart infusion agar and nutrient agar plates for *E. hirae* and *E. coli,* respectively, and incubated at 37 °C for 24 h. Viable cells in these culture plates were enumerated by colony counting method (Vilela et al. 2012) and PS inactivated bacteria were further confirmed by flow cytometry as detailed under “Flow cytometry analysis”.

The percentage inactivation of microorganisms from each duplicate runs in the study was calculated as per the following equation and the results shown are average of two values:

1 $$\% \, {\text{inactivation}} = \frac{{C_{\text{i}} - C_{\text{f}} }}{{C_{\text{i}} }} \times 100$$

where *C*_i_ and *C*_f_ are the initial and final viable cell counts.

Statistical analysis in the form of analysis of variance (ANOVA) and student ‘*t*’ test was carried out to validate the roles played by different parameters and their interactions on the bacterial photo-inactivation. All these statistical analyses were performed using the software MINITAB (version 16, PA, USA).

### Flow cytometry analysis

PS treated bacterial cells from the previous experiments were obtained by centrifuging the cell suspension at 10,000×*g* for 10 min and the pellet was washed with PBS of a suitable pH. Later, the pellet was suspended in 1 ml of PBS to which 20 µL of propidium iodide (PI) solution (prepared by dissolving in distilled water in the ratio 1:1) was added and incubated in dark for 15 min. After which, 100,000 cells from the sample obtained were analyzed using BD FACS Calibur™, USA flow cytometer equipped with an argon laser (L1) (wavelength 488 nm; Fluorescence channel: FL-2 yellow). PI fluorescence was measured to discriminate between live and dead cells. Flow cytometry analysis is done to simulate and support the results of colony counting method.

### Confirming reactive oxygen species (ROS) generation

The bacterial cell suspension in respective pH phosphate buffered saline and dilutions as described in Table 2 were added with DCFDA (dihydrochlorofluorescin diacetate) 20 μL of 20 μM concentration and incubated for 30 min at 37 °C, then the suspension was added with photosensitizer and kept in dark on a gel rocker for 30 min. Later, it was exposed to visible light or UV-A light for photosensitizers MB and SAQS, respectively.

10 μl of the suspension after light period was spread on agar plates for viable cell count and 1 mL of the suspension was checked for DCF (2, 7 dichlorofluorescin) fluorescence by exciting at 488 nm and emission spectra studied over 510–540 nm using Fluoromax 4.

### Measurement of lipid peroxidation as an index of oxidative stress

The bacterial cell suspension was prepared and the inactivation experiments were carried out as described in “Inactivation experiments”. Treated bacterial cells were obtained in the form of pellet and lipid peroxidation products of cell lysate were determined as thiobarbituric acid reactive substances (Trivedi et al. 2005). Bacterial pellet was resuspended in PBS of respective pH and sonicated by keeping on ice using probe sonicator. An aliquot (100 μL) of bacterial lysate was allowed to react with 10 %, 4 °C trichloroacetic acid (200 μL) for 15 min on ice. Later, the suspension was centrifuged at 3000×*g* for 15 min at 4 °C to get the supernatant which is made to react with thiobarbituric acid in equal volumes by placing in boiling water bath for 10 min. After cooling, its absorbance was taken at 532 nm to determine thiobarbituric acid reactive substances using tetraethoxy propane as standard.

### Measurement of protein carbonyl as an index of oxidative stress

Inactivation experiments were performed as mentioned in “Inactivation experiments”. The pellets of treated bacterial cells were washed with PBS and lysed by probe sonicator keeping on ice. Lysate was divided equally in two parts and were added with equal volume of 10 % trichloroacetic acid at 4 °C. The mixture was incubated for 15 min at 4 °C and then centrifuged at 3000×*g* for 15 min. The precipitate obtained in one half was added with 500 μL of 0.2 % DNPH in 2 N HCl and other half was added with 500 μL of 2 N HCl. The mixtures were then incubated at 37 °C for 1 h with continuous vortexing then added with 55 μL of 100 % TCA for precipitating protein. Samples were centrifuged and pellet was washed with a mixture of ethanol and ethylacetate later dissolved in 600 μL of 6 M guanidine hydrochloride. The final mixture was incubated for 30 min and the absorbance recorded at 370 nm (Castegna et al. 2003).

## Results

### Photo-inactivation of *E. hiraeand E. coli*

**Fig. 1:**
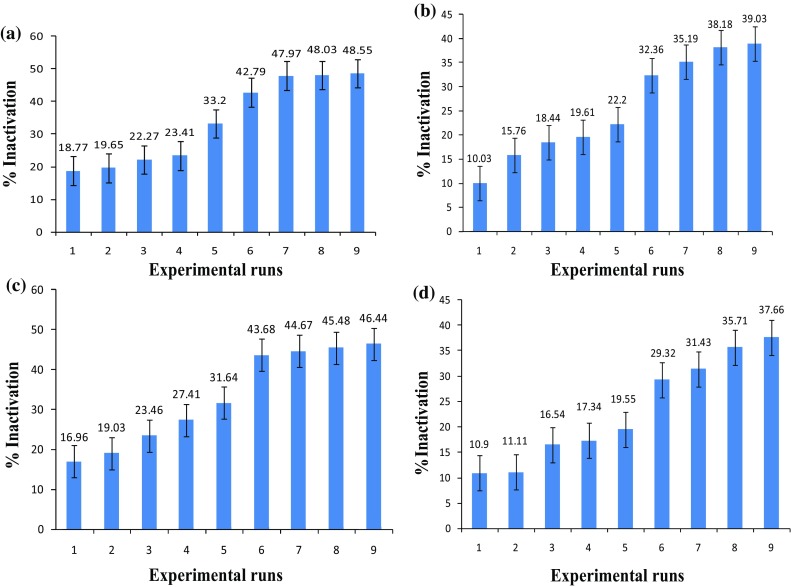
Photo-inactivation of *E. hirae* and *E. coli* obtained in the different experimental runs using MB in presence of white light and SAQS in presence of UV-A light at 30-min dark incubation period **a** *E. hirae* and MB, **b** *E. hirae* and SAQS, **c** *E. coli* and MB, **d** *E. coli* and SAQS

Photo-inactivation efficiency for *E. hirae* and *E. coli* using MB varied in the range 18.77–48.55 and 16.96–46.44 %, respectively (Fig. 1). In case of photo-inactivation using SAQS the inactivation efficiency varied in the range 10.03–39.03 % for *E. hirae* and 10.90–37.66 % for *E. coli,* which are lower as compared with the efficiency obtained due to MB (Fig. 1). No significant change in CFU was observed in dark controls.

The photo-inactivation efficiency of the bacteria increased with an increase in the PS concentration and a higher dilution of the bacteria (i.e., 1000 times initial viable cell count). Increase in pH of the cell suspension also showed significant effect on the photo-inactivation but depended on the combination levels of the other two factors (PS initial concentration and initial viable cell count) (Fig. 4). In addition, incubation in dark prior to the light exposure showed significant differences in the inactivation efficiency (data not shown). An increase in the inactivation efficiency for both the bacteria due to the dyes was observed with an increase in the dark incubation period with a maximum inactivation efficiency obtained at 30-min dark incubation time.

**Fig. 2:**
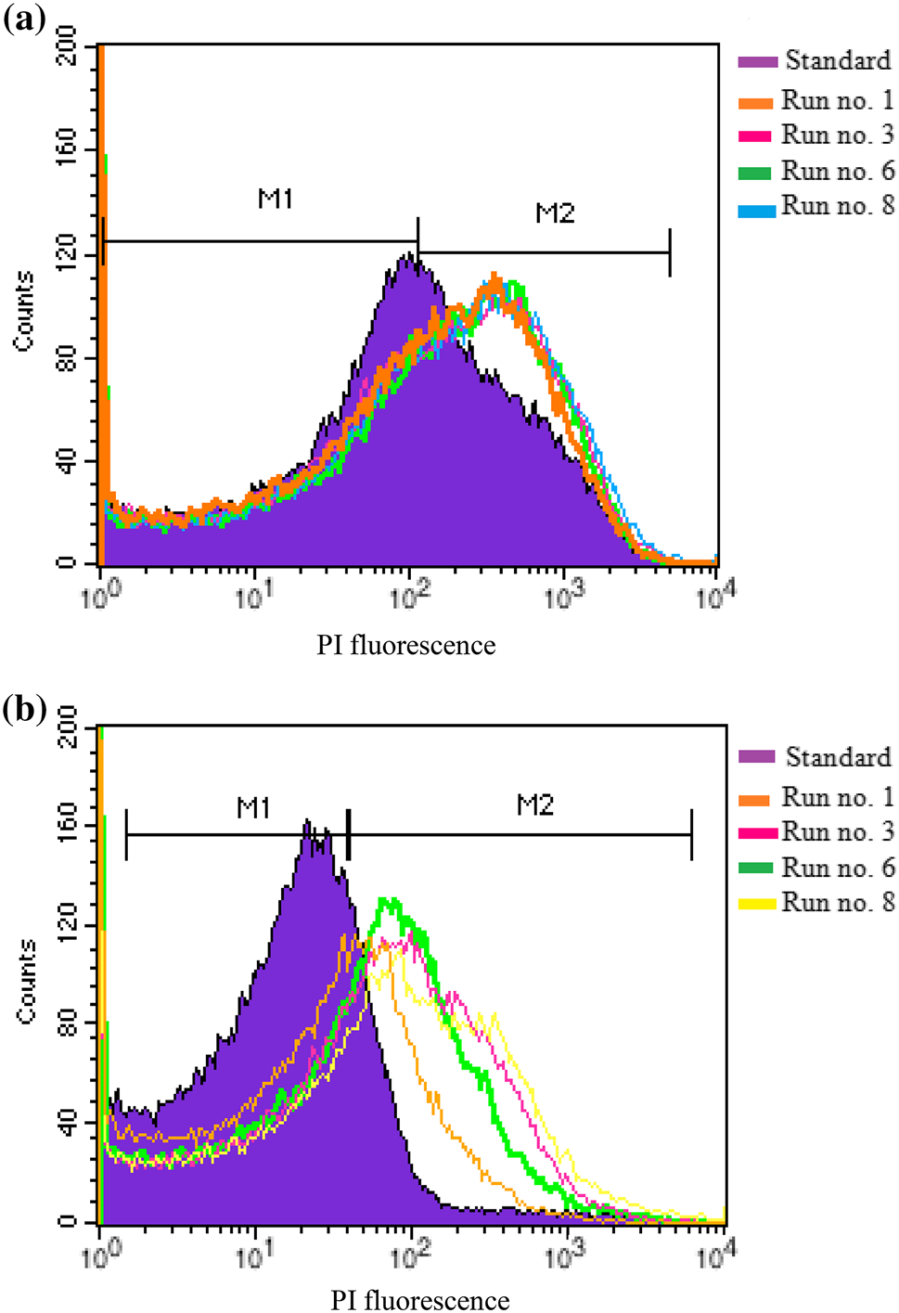
Flow cytometry histograms obtained for photo-inactivation of *E. hirae* using **a** MB, **b** SAQS (sample dilution = 10 times, dark incubation period for photo-inactivation = 30 min)

**Fig. 3:**
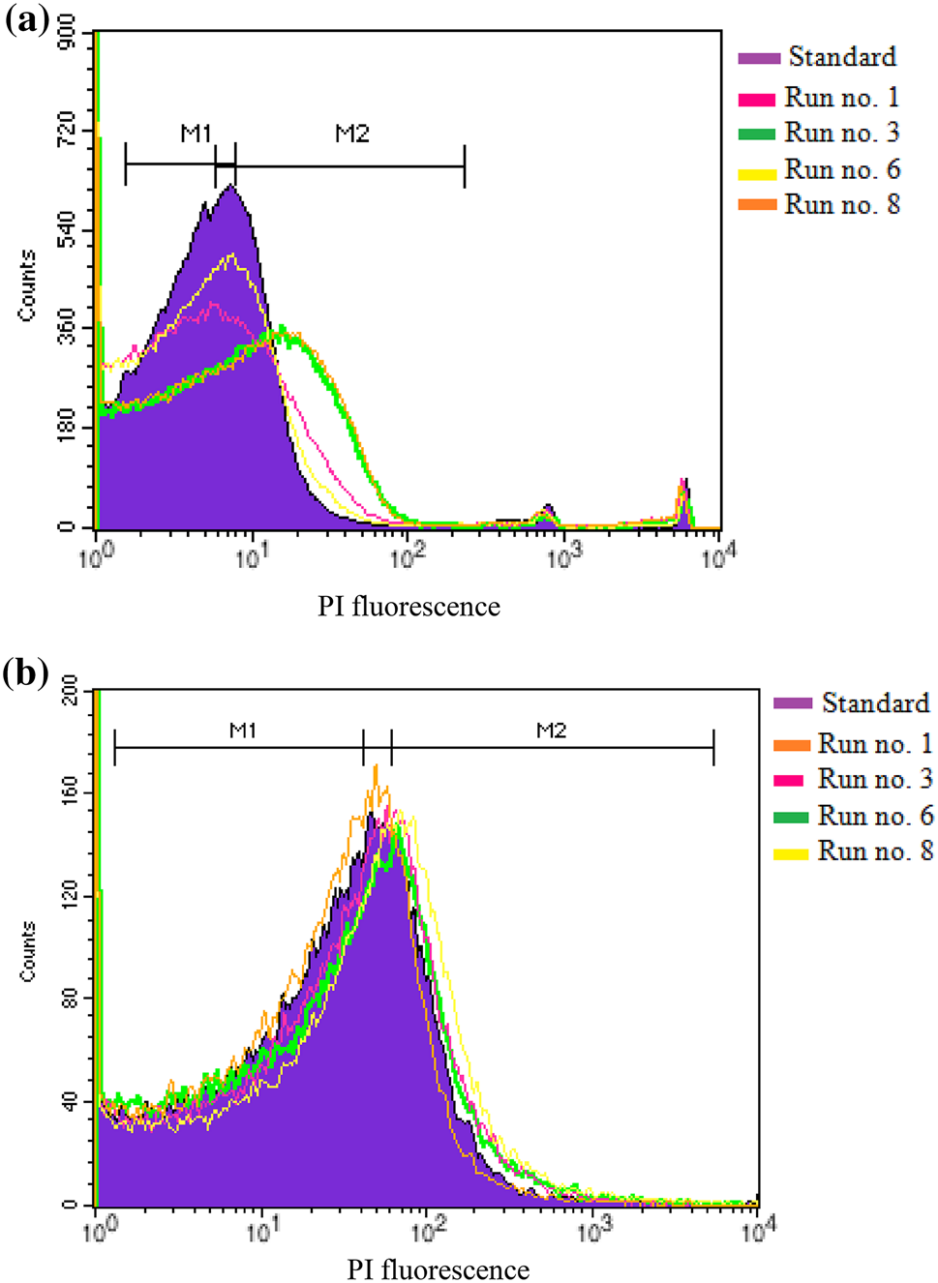
Flow cytometry histograms obtained for photo-inactivation of *E. coli* using **a** MB, **b** SAQS (sample dilution = 10 times, dark incubation period for photo-inactivation = 30 min)

The validity of the cell viability assay performed using the colony counting method was verified both qualitatively and quantitatively by flow cytometry (Figs. 2, 3). Shift in fluorescence peaks of propidium iodide is observed with an increase in the PS initial concentration for 30-min dark incubation time. In these histograms, gates M1 and M2 are defined in accord with the control sample (i.e., bacterial suspension without treatment with MB or SAQS); hence, the area under M_1_ depicts the live cells whereas the area under M_2_ represents dead cells. Statistical analyses of M_1_ and M_2_ (Table 3) further revealed that photo-inactivation of *E. hirae* by both MB and SAQS is better than that of *E. coli*.

**Table 3:** Percent live and dead cells obtained from the cell cytometry analysis data for photo-inactivation of bacteria using (a) MB (b) SAQS

*E. hirae*			*E. coli*	
Run no.	M_1_ (%)	M_2_ (%)	M_1_ (%)	M_2_ (%)
(a)				
Standard	49.91	39.95	48.80	31.48
1	36.78	48.27	37.16	42.61
3	32.61	49.88	37.97	46.35
6	35.48	50.39	30.64	48.47
8	32.96	51.78	30.27	48.99
(b)				
Standard	66.52	31.61	64.99	17.04
1	61.73	34.46	30.69	30.69
3	55.90	37.33	29.25	49.71
6	56.06	39.27	27.62	54.00
8	49.95	44.27	25.63	54.68

### Statistical analysis

For a better understanding of the role of different variables on the inactivation of *E. coli* and *E. hirae*, statistical analysis of the results in the form of analysis of variance (ANOVA) and student ‘t’ test was performed.

The ANOVA of photo-inactivation results obtained at 30-min dark incubation period shows a high Fischer’s ‘*F*’ value (66.83 and 235.75 for *E. hirae* whereas 169.77 and 153.50 for *E. coli* with MB and SAQS, respectively) and a low probability ‘*P*’ value of the regression model (*P* = 0.00) for *E. hirae* and *E. coli* with MB and SAQS, indicates its validity in explaining the variations in the results. Further, the results suggest that individual parameter effects and 2-way interaction effects due to the viable cell count were statistically significant. Accuracy and precision of the models, in the form of determination coefficient (*R* ^2^) (*R* ^2^ = 91.98 and 96.66 for *E. hirae* and *E. coli* with MB whereas *R* ^2^ = 98.76 and 98.13 for *E. hirae* and *E. coli* with SAQS), adjusted *R* ^2^ (88.42 and 95.17 for *E. hirae* and *E. coli* with MB, 97.66 and 96.46 for *E. hirae* and *E. coli* with SAQS), standard deviation (SD) (18.02 and 12.47 for *E. hirae* and *E. coli* with MB, 12.68 and 9.23 for *E. hirae* and *E. coli* with SAQS) and predicted residual error sum of squares (PRESS) (13,134.1 and 6739.20 for *E. hirae* and *E. coli* with MB, 6381.69 and 3315.16 for *E. hirae* and *E. coli* with SAQS) suggest that the models were highly efficient in predicting the experimental photo-inactivation results.

The estimated coefficients of individual and interaction effects between the variables as well confirmed these results. A highly significant effect of initial PS concentration (*P* = 0.000), initial viable cell count (*P* = 0.000) and combined effect of PS initial concentration and initial viable cell count (*P* = 0.028) on *E. hirae* inactivation using MB is observed. In the case of *E. coli* inactivation using MB, significant effect is observed due to the PS concentration (*P* = 0.000), pH of the suspension (*P* = 0.001), initial viable cell count (*P* = 0.000) and combined effect of initial PS concentration and initial viable cell count (*P* = 0.026); however, the other interaction effects between the variables were found insignificant. Similarly, the student ‘*t*’ test results revealed significant effect of PS initial concentration (*P* = 0.000), pH of the suspension (*P* = 0.009 for *E. hirae* and *P* = 0.002 for *E. coli*), initial viable cell count (*P* = 0.000) and combined effect of PS initial concentration and initial viable cell count (*P* = 0.020 for *E. hirae* and *P* = 0.025 for *E. coli*) on the inactivation of *E. hirae* and *E. coli* using SAQS.

**Fig. 4:**
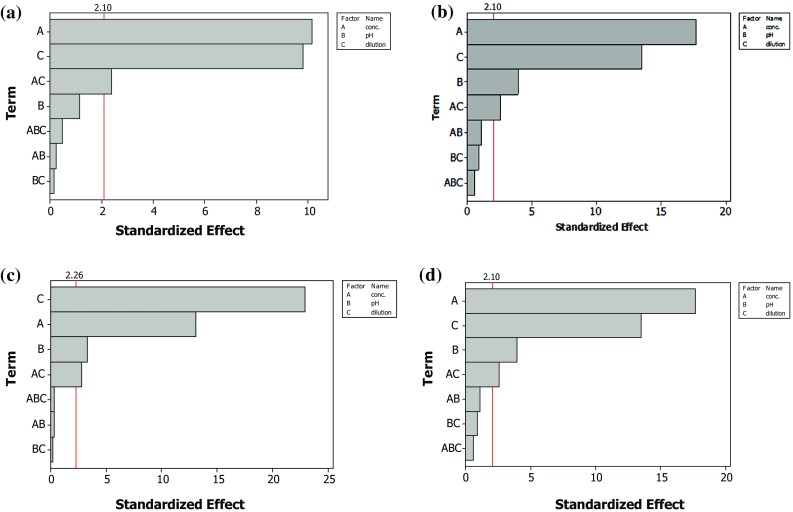
Pareto chart showing the effect of different variables on photo-inactivation of bacteria using MB in presence of white light and SAQS in presence of UV-A light **a** *E. hirae* and MB, **b** *E. coli* and MB, **c** *E. hirae* and SAQS, **d** *E. coli* and SAQS (dark incubation period = 30 min)

All these results of effect of variables on the photo-inactivation of *E. hirae* and *E.coli* are depicted in a better way in the form of pareto charts and are illustrated in Fig. 4. Horizontal bars in these charts represent effects (i.e., individual and interaction terms) of the parameters and the effects which extend past the reference line (vertical line on the chart) denote the significant ones (*α* = 0.05).

### Reactive oxygen species (ROS) confirmation and action

**Fig. 5:**
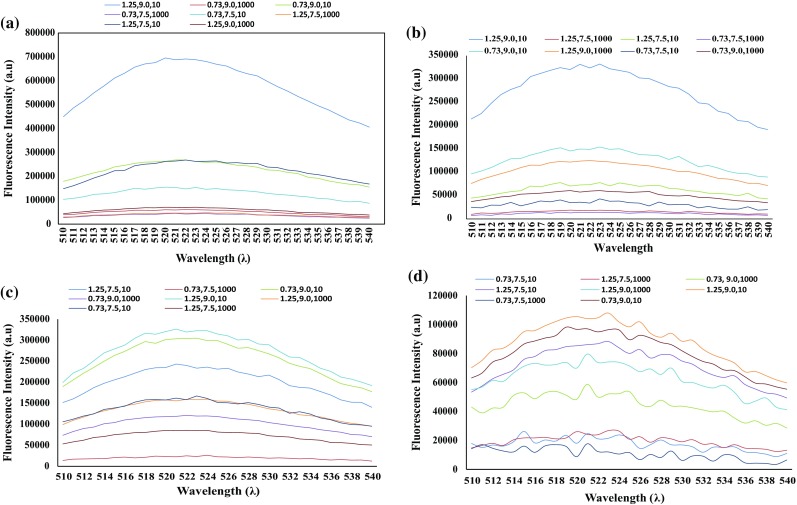
Fluorescence curves for different experimental run in case of **a** *E. hirae* + MB, **b** *E. coli* + MB, **c** *E. hirae* + SAQS, **d** *E. coli* + SAQS

ROS is measured as explained in “Confirming reactive oxygen species (ROS) generation” and the results revealed that experimental run number 8 (as per the design in Table 2) gives the maximum fluorescence in bacterial suspension and hence it can be concluded that maximum ROS is generated in this experimental run. From Fig. 5, it is clear that maximum ROS production occurs with methylene blue exposed to visible light as compared to experiments with SAQS exposed to UV-A light.

Lipid peroxidation and protein carbonylation index (Table 4) shows occurrence of oxidative degradation of lipids and proteins present in the membrane. The obtained results showed high lipid peroxidation and protein carbonylation levels for *E. hirae* and *E. coli* when treated with MB as compared to when treated with SAQS.

**Table 4:** Results of lipid peroxidation and protein carbonyl assay using (a) MB (b) SAQS

Process parameters		Lipid peroxidation		Protein carbonylation	
pH	Conc. of PS (μmol/L)	*E. coli*	*E. hirae*	*E. coli*	*E. hirae*
(a)					
7.3	0	3.51	3.63	64.21	69.11
7.5	0.73	5.93	7.26	67.95	91.46
7.5	1.25	7.25	7.41	74.36	95.91
9.0	0.73	7.37	8.09	68.16	94.70
9.0	1.25	7.96	9.36	84.99	98.40
(b)					
7.3	0	2.08	2.16	31.24	27.43
7.5	0.73	3.15	3.51	36.23	33.09
7.5	1.25	3.52	3.76	40.26	35.55
9.0	0.73	3.73	3.84	36.51	34.98
9.0	1.25	3.85	4.09	43.18	45.71

## Discussion

### Mechanism of photo-inactivation

The decrease in bacterial cell viability due to the cationic photo-sensitizers (MB and SAQS) is attributed to the positive charge of these molecules that favors its binding at critical cellular sites which once damaged by exposure to light causes the inactivation of both Gram positive and Gram negative bacteria (Jori and Brown 2004). It has been reported that positive charge on PS promotes electrostatic interaction with negatively charged sites at the outer surface of the bacterial cells which increases their photo-inactivation efficiency (Caminos et al. 2008). However, the results obtained (Fig. 1) showed that both the cationic photo-sensitizers (MB and SAQS) are more efficient against *E. hirae* than *E. coli.* This is mainly attributed to the presence of outer lipopolysaccharide layer in Gram negative bacteria such as *E. coli* that helps the bacteria avoid or limit the uptake of the photo-sensitizers and/or the reactive oxygen species produced by these compounds thereby escaping from the photo-inactivation process (Dahl et al. 1989; Ergaieg and Seux 2009).

The photo-inactivation results were further confirmed by flow cytometry analysis of the PS treated bacteria. As propidium iodide is not permeable through the intact cell membrane, it only gets internalized and binds to DNA of the cells whose membranes are compromised. Hence, the shift in the fluorescence peaks of propidium iodide added with PS treated bacteria from the standard peak represented the fact that the cell membrane in these bacteria was damaged thereby leading to their inactivation.

### Effect of parameters

PS initial concentration strongly influenced the photo-inactivation of microorganisms (Tables 3, 4), suggesting that the effect is due to either an increase in the quantum yield of reactive oxygen species (Ergaieg and Seux 2009) or profound interaction of the PS with bacterial surface or both (Jori and Brown 2004). pH, on the other hand, showed its significant effect only at a higher level (alkaline pH) and at a prolonged incubation period (30 min) in dark. Chen et al. (2011) reported that MB is more effective under basic pH than under acidic condition probably due to its transition between singlet and triplet states. The effect of pH was not significant for a short dark incubation period of 5 min probably due to insufficient contact time for interaction between the PS and the bacteria. Compared with initial PS concentration and pH, dilution is easily correlated with the initial bacterial count in the suspension, and, therefore, as dilution increases, the number of bacterial cells in the suspension decreases yielding a better interaction with PS at the same concentration as compared with less dilute suspension of the bacteria. Also, the chance of encountering reactive oxygen species increases when the dilution is high, which further enhances the photo-inactivation efficiency of the bacteria. Similar results were reported in the literature but for photocatalysis of *E. coli* using TiO_2_ (Benabbou et al. 2007).

The enhanced photo-inactivation efficiency with an increase in the dark incubation period is attributed to an increase in the contact time between the bacteria and the PS at prolonged dark incubation as Ergaieg and Seux (2009) reported that positive charge on the photosensitizer molecule allows it to bind or in some cases penetrate into the microbial cell and the photosensitizers used in this study are cationic.

To gain further insight in the mechanism of photoinactivation ROS measurement was done which showed direct relationship between inactivation and ROS production. Evidences have been presented in the form of lipid peroxidation and protein carbonyl index which suggests that the ROS generated during the reaction acts on the membrane of the bacteria and alters the lipid and protein of the membrane to form reactive aldehydes and/or ketones resulting in cell damage.

Table 5 compares the photoinactivation results obtained in this study with those reported in the literature, which clearly reveals very good potential of the dye sensitized photoinactivation method for water disinfection. However, to achieve the best results the photoinactivation process parameters need to be optimized. In addition, its efficiency against a wide range of microorganisms needs to be tested and the residual PS dye remaining in the treated water/wastewater need to be efficiently removed prior to its use/discharge.

**Table 5:** Comparison of photo-inactivation results obtained in this study with those reported in the literature

Organism used	Photo-inactivation results	References
*Enterococcus hirae* and *Escherichia coli*	Photo-inactivation rate constant *K*_*E. hirae*_ = 1.517 min^−1^ *K*_*E. coli*_ = 0.513 min^−1^ with porphyrin (TMPyP)	Ergaieg and Seux (2009)
3 Gram positive and 5 Gram negative bacteria	Inactivation of Gram +ve and Gram –ve bacteria was 3–25 times higher at pH 9 than at pH 5	Chen et al. (2011)
*Enterococcus faecalis* and *Escherichia coli*	100 % for *E. faecalis* and 45.71 % for *E. coli* with porphyrin Di-Py^+^-Me-Di-CO_2_H	Alves et al. (2009)
*Escherichia coli*	81.9 % with 20 μmol/L of MB incorporated in ZSM-5 zeolite channels under red LED light	Smolinska et al. (2010)
*Escherichia coli* and *Enterococcus hirae*	48.55 % for *E. hirae* and 46.44 % for *E.coli* with 1.25 μmol/L MB Using SAQS, 39.03 % inactivation of *E. hirae* and 37.66 % inactivation of *E. coli*	Present study

## Conclusions

The results obtained in this study revealed that both cationic photosensitizers are efficient against Gram positive and Gram negative bacterial strains, with a higher efficiency against the Gram positive *E. hirae.* Between the two PS, MB was more efficient against both the bacterial strains than SAQS at the same concentration, pH and viable cell count values. Cell cytometry analysis further revealed that the mechanism of photo-inactivation involved bacterial cell membrane damage by the PS. Statistical analysis of the results revealed that besides the significant individual effect due to concentration of PS, pH of bacterial suspension and dilution, interaction effect between concentration of PS and initial viable cell count was significant for the bacterial inactivation.
